# Impact of delayed elective urological surgery: A prospective observational study

**DOI:** 10.1002/bco2.70197

**Published:** 2026-04-11

**Authors:** Iva Simunovic, Hanna Zurl, Julia Altziebler, Conrad Leitsmann, Lukas Scheipner, Johannes Mischinger, Anna Mangge, Klara Pohl, Almut Frank, Alexandra Weiss, Gerhard Bachmaier, Sascha Ahyai, Marianne Leitsmann

**Affiliations:** ^1^ Department of Urology, University Hospital Graz Medical University of Graz Graz Austria; ^2^ Center of Surgery and Public Health, Brigham and Women's Hospital Harvard Medical School Boston Massachusetts USA; ^3^ Medizinische Versorgungsplanung, University Hospital Graz Graz Austria; ^4^ Department of Medical Psychology, University Hospital Graz Graz Austria; ^5^ Institute for Medical Informatics, Statistics and Documentation Medical University of Graz Graz Austria; ^6^ Institute for Applied Quality Improvement and Research in Health Care Göttingen Germany

**Keywords:** burden, complications, elective surgery, mental health, postponement, waiting times

## Abstract

**Objectives:**

This study aimed to evaluate the psychological and physical impact of delayed elective urological surgeries, as limited surgical capacity has led to frequent cancellations and prolonged waiting times.

**Patients and Methods:**

Between July 2023 and September 2025, patients admitted for elective surgery completed questionnaires including the NCCN Distress Thermometer, Severity Symptom Scale (SSS), PROMIS‐10 and items on delay‐related complications. Primary outcomes were psychological distress (NCCN ≥4), symptom burden (SSS ≥ 3) and PROMIS‐10 Global Health T‐scores.

**Results:**

A total of 488 patients were analysed: 183 (37.5%) with confirmed/suspected oncological diagnoses and 304 (62.3%) non‐oncological. Surgery was postponed in 51% of cases. Mean waiting time was 23.9 weeks (SD ± 19.6), longer for postponed patients (31.8 vs. 15.5 weeks; *p* < 0.001) and more frequent among non‐oncological patients (64.5% vs. 28.4%; *p* < 0.001). Postponed patients reported higher symptom burden (SSS ≥ 3: 40.6% vs. 30.1%; *p* = 0.03) and lower Global Mental Health T‐scores (43.9 ± 5.6 vs. 45.1 ± 5.3; *p* = 0.03). Regression analyses showed patients in the third and fourth waiting‐time quartiles had higher SSS scores compared with the shortest quartile (*β* = 0.36, *p* = 0.02; *β* = 0.50, *p* = 0.001; overall model *p* = 0.01). Among postponed patients, 30.1% reported complications, most commonly pain (12.9%), urinary tract infection (11.7%), urinary retention (6.4%) and macroscopic haematuria (2.4%).

**Conclusion:**

Prolonged waiting times and surgical postponements contribute to physical complaints and psychological distress. Persistent nursing shortages constrain capacity, emphasizing the need for long‐term structural planning. Strengthening resources while maintaining patient‐centred care is essential to prevent avoidable harm in elective urological surgery.

## 1 INTRODUCTION

During the COVID‐19 pandemic and its associated measures most elective procedures were postponed for months, causing ongoing delays and straining the healthcare system.^1^, ^2^ In countries such as Austria, this trend persisted beyond the pandemic, largely due to a persistent shortage of medical personnel, particularly among nursing staff. As a result, operating theatres are frequently subject to short‐notice closures, leading to surgical delays and prolonged waiting times for elective procedures.^3^

Delayed treatment is well‐documented to have negative effects on the prognosis of many urological tumours.^4^, ^5^ Because of this, the shortage of medical resources has compelled departments to prioritize uro‐oncological procedures. In contrast, a significant number of postponed or cancelled procedures for non‐oncological indications such as benign prostatic syndrome (BPS) and other benign urological disorders have received comparatively little attention. While the immediate focus has rightly been on addressing delays in cancer treatment, the long‐term consequences of deferred care for benign conditions must not be overlooked. Over the coming years, we are likely to witness not only the effect of postponed cancer diagnoses and therapies^6^ but also the growing burden of complications, impaired quality of life and increased healthcare needs resulting from delayed or missed interventions for benign urological conditions. This underscores the importance of addressing the broader impact of resource shortages on all aspects of urological care.

Currently little is known about the psychological and physical impact of postponing elective operations in patients with urological conditions, particularly in patients with benign conditions. We hypothesize that extended waiting times and cancelled surgeries are associated with significant burden and adverse outcomes in affected patients.

## 2 PATIENTS AND METHODS

### 2.1 Study population

This unicentric prospective observational study was conducted at the Department of Urology, Medical University of Graz. It was approved by the local ethics committee (35‐322 ex 22/23) and registered at ClinicalTrials.gov (NCT06067373). All patients aged 18 years or older who were admitted for elective urological surgery between July 2023 and September 2025 were eligible for inclusion. Patients were consecutively invited to participate in the study at the time of admission for surgery. After providing written informed consent, participants completed a 12‐item paper‐based questionnaire including validated survey instruments and study‐specific questions.

### 2.2 Data collection

At enrollment, we documented patient characteristics, including age, gender, diagnosis (suspected or confirmed malignancy vs. no malignancy) and the type of planned surgery. Furthermore, we recorded the date of surgery registration and the actual surgery date.

Using a 12‐item paper‐based questionnaire, patients were asked about their waiting time for surgery, whether their surgery was postponed, and if applicable, the number of postponements. Most postponements occurred prior to the scheduled surgery date and were communicated in advance. In cases of postponement, patients were asked whether the reason was known. Possible reasons included institutional capacity constraints (e.g., lack of operating room slots), patient‐related factors (e.g., personal request) and medical issues (e.g., health reasons and incomplete preoperative preparation). Employment status was also recorded. Furthermore, we assessed postponement‐related complications, unplanned physician visits and readmissions, hospital admissions, emergency surgeries and impact on professional activity.

Self‐reported waiting times and the number of postponed surgeries, as well as relevant clinical data, including complications and interventions, were verified using medical records and institutional surgery schedules.

### 2.3 Outcomes

The primary aim of the study was to assess the psychological and physical burden on patients associated with surgical postponement in urological patients. Psychological burden was assessed using the National Comprehensive Cancer Network (NCCN) Distress Thermometer and the Global Mental Health T‐score. Physical burden was assessed using the Severity Symptom Scale (SSS) and the PROMIS Global Physical Health T‐score.^7^ We chose these validated, broadly applicable instruments due to the heterogeneous nature of our patient cohort, which included individuals with both oncological and non‐oncological conditions.

The NCCN Distress Thermometer is a single‐item, self‐report measure of psychological distress ranging from 0 (*No distress*) to 10 (*Extreme distress*). A score of ≥4 indicates clinically meaningful distress.

The SSS is a standardized self‐administered tool used to assess the severity of symptoms experienced by patients. SSS scores range from 1 (*No symptoms*) to 5 (*Very severe symptoms, unable to carry out daily activities*).

Health‐related quality of life was measured using the German version of the 10‐item PROMIS Global Health scale^8^ which includes a 5‐point Likert scale assessing physical function, pain, fatigue, emotional well‐being, social health and general perception of health. From these responses, two T‐scores are calculated: Global Physical Health and Global Mental Health T‐score.

We also assessed complications among patients with postponed surgery. We focused on pain, urinary tract infection (UTI), urinary retention and macroscopic haematuria as key complication endpoints, as these are among the most frequently observed adverse events in urological practice. They often lead to emergency visits or unplanned physician consultations. Pain, in particular, is a highly relevant patient‐reported outcome that often prompts unplanned medical contact and reflects a significant symptom burden, especially in patients with catheter use or recurrent infections.^9^ Tumour progression was defined as histopathological upstaging of the initial preoperative clinical TNM staging (based on computed tomography/magnetic resonance imaging) compared to the histopathological findings after surgery, in the context of delayed surgery. Other potential complications were not systematically captured.

### 2.4 Statistical methods

Descriptive statistics were used to summarize baseline characteristics, with categorical variables reported as frequencies and proportions and continuous variables as means and standard deviations (SD). Patient characteristics were compared between those who underwent surgery as scheduled and those whose surgery was postponed, using chi‐squared tests for categorical variables and Wilcoxon rank‐sum tests for continuous variables.

To compare psychological and physical burden between patients with postponed surgery and those without, Wilcoxon rank‐sum tests and *t*‐tests were applied for continuous outcomes (Global Mental Health T‐scores and Global Physical Health T‐scores). Results from the NCCN Distress Thermometer and the SSS were dichotomized as previously described (NCCN: <4 vs. ≥4; SSS: <3 vs. ≥3), and groups were compared using chi‐squared tests.^10^, ^11^

Binary logistic regression was used to assess the relationship between waiting time for the surgery and cutoffs of physical impact (SSS score <3 vs. ≥3) and psychological impact (NCCN score <4 vs. ≥4).

To assess further the impact of waiting time duration on physical and psychological outcomes, regression analyses were performed based on waiting time divided into quartiles.

In an additional subgroup analysis, outcomes were compared between patients with delayed oncological surgery and delayed non‐oncological surgery. Furthermore, multivariable analyses adjusting for waiting time were performed.

All analyses were conducted using the Stata version 17.0 BE‐Basic Edition (StataCorp, College Station, TX). Two‐sided significance tests were applied, with *p*‐values less than 0.05 considered statistically significant.

## 3 RESULTS

Between July 2023 and September 2025, a total 488 Patients admitted to our department for elective surgery were analysed. Baseline characteristics are summarized in Table 1. The mean age was 64.0 (SD ± 13.9) and 84.6% (*n* = 413) were male; 183 (37.5%) patients had a suspected or confirmed oncological diagnosis, and 304 (62.3%) did not. The most common procedures were endoscopic surgeries for benign prostatic obstruction (transurethral resection of the prostate ‐ TURP/holmium laser enucleation of the prostate—HoLEP; 38.9%), transurethral resection of bladder tumour (TURBT; 16.6%) and reconstructive surgeries (11.1%). Procedures most frequently affected by postponement were TURP (72.7% of all performed TURP procedures were postponed at least once), HoLEP (63.8%), reconstructive surgery (66.7%) and interventions for urolithiasis such as ureterorenoscopy/percutaneous nephrolitohotomy (50.0%). In contrast, oncological procedures were less often postponed, including radical prostatectomy, TURBT and (partial) nephrectomy. Overall, 25.2% of the cohort were employed at the time of surgery.

**TABLE 1:** Baseline characteristics of the study cohort stratified according to surgery postponement status.

	Overall	Surgery not postponed	Surgery postponed	*p*‐value
Total (*n*, %)	488 (100.00)	239 (48.98)	249 (51.02)	
Age, year (mean ± SD)	64.03 (13.87)	63.97 (14.09)	64.10 (13.68)	0.86
Sex (*n*, %)				0.28
*Male*	413 (84.63)	198 (47.94)	215 (52.06)	
*Female*	75 (15.37)	41 (54.67)	34 (45.33)	
Surgery indication (*n*, %)				**<0.001**
*(Suspected) malignancy*	183 (37.50)	131 (71.58)	52 (28.42)	
*No malignancy*	304 (62.30)	108 (35.53)	196 (64.47)	
*NA*	1 (0.20)	0	1 (100.00)	
Surgery (*n*, %)				
*TURP*	110 (22.54)	30 (27.27)	80 (72.73)	
*TURBT*	81 (16.60)	59 (72.84)	22 (27.16)	
*HoLEP*	80 (16.39)	29 (36.25)	51 (63.75)	
*Reconstructive surgery*	54 (11.07)	18 (33.33)	36 (66.67)	
*URS*	30 (6.15)	15 (50.00)	15 (50.00)	
*Partial nephrectomy*	28 (5.74)	17 (60.71)	11 (39.29)	
*Radical prostatectomy*	25 (5.12)	21 (84.00)	4 (16.00)	
*External genitalia surgery*	18 (3.69)	10 (55.56)	8 (44.44)	
*Nephrectomy*	13 (2.66)	9 (69.23)	4 (30.77)	
*Cystectomy*	12 (2.46)	8 (66.67)	4 (33.33)	
*PCNL*	8 (1.64)	3 (37.50)	5 (62.50)	
*Semicastration*	4 (0.82)	3 (75.00)	1 (25.00)	
*Adrenalectomy*	2 (0.41)	1 (50.00)	1 (50.00)	
*Nephroureterectomy*	2 (0.41)	2 (100.00)	0	
*Others*	21 (4.30)	14 (66.67)	7 (33.33)	
Employment status (*n*,%)				0.56
*Not employed/retired*	342 (70.08)	159 (46.49)	183 (53.51)	
*Employed*	123 (25.20)	61 (49.59)	62 (50.41)	
*NA*	23 (4.71)	19 (82.61)	4 (17.39)	
Waiting time (mean ± SD)	23.85 (19.64)	15.54 (12.67)	31.76 (21.75)	**<0.001**
Number of postponements (*n*, %)				
*0*		239 (48.98)		
*1*			150 (30.74)	
*2*			63 (12.91)	
*3*			19 (3.89)	
*4*			3 (0.61)	
*5*			2 (0.41)	
*6*			1 (0.20)	
*NA*			11 (2.25%)	

Abbreviations: HoLEP, holmium laser enucleation of the prostate; NA, not available; PCNL, percutaneous nephrolithotomy; SD, standard deviation; TURBT, transurethral resection of bladder tumor; TURP, transurethral resection of the prostate; URS, ureteroscopy.

The overall mean waiting time for surgery was 23.9 weeks (SD ± 19.6). Patients whose surgery was not postponed waited 15.5 weeks (SD ± 12.7), while those with postponements waited 31.8 weeks (SD ± 21.8, *p* < 0.001). Postponement was significantly more common among patients with non‐oncological diagnosis (64.5% vs. 28.4%, *p* < 0.001). The mean waiting time for patients with a non‐oncological diagnosis was 30.6 weeks (SD ± 20.5), compared to 12.8 weeks (SD ± 11.7) in patients with a suspected or confirmed oncological diagnosis.

Surgery was postponed at least once in 249 (51.0%) patients. The most frequently reported reason was institutional: lack of operating room capacity (44.6%), followed by health reasons (8.4%) and incomplete surgical preparation (4.8%). Only a small proportion (3.2%) of delays occurred at the patient's own request.

### 3.1 Psychological distress

The median NCCN score at the admission for surgery across the cohort was 4 (IQR 2–6) with no difference between patients with or without surgery postponement. The mean Global Mental Health T‐score across the cohort was 44.5 (SD ± 5.4). Patients whose surgeries were postponed showed significantly lower Global Mental Health T‐scores than those whose surgeries were not postponed (mean 43.9 [SD ± 5.6] vs. 45.1 [SD ± 5.3], *p* = 0.03; Table 3).

Longer waiting times were not associated with higher odds of experiencing significant psychological distress (NCCN score ≥4) (OR 1.01, 95% CI: 1.0–1.02; *p* = 0.23) or lower Global Mental Health T‐score (OR 1.00, 95% CI: 0.99–1.01; *p* = 1.0) prior to surgery.

### 3.2 Physical impact and symptom burden

The proportion of patients with moderate to very severe symptoms (SSS score ≥3) was significantly higher among patients whose surgery was postponed compared to patients whose surgery was done as scheduled (40.6% vs. 30.1%, *p* = 0.03). Overall, the Global Physical Health T‐score was 43.0 (SD ± 5.3) and did not significantly differ between the two groups (Table 3).

**FIGURE 1:**
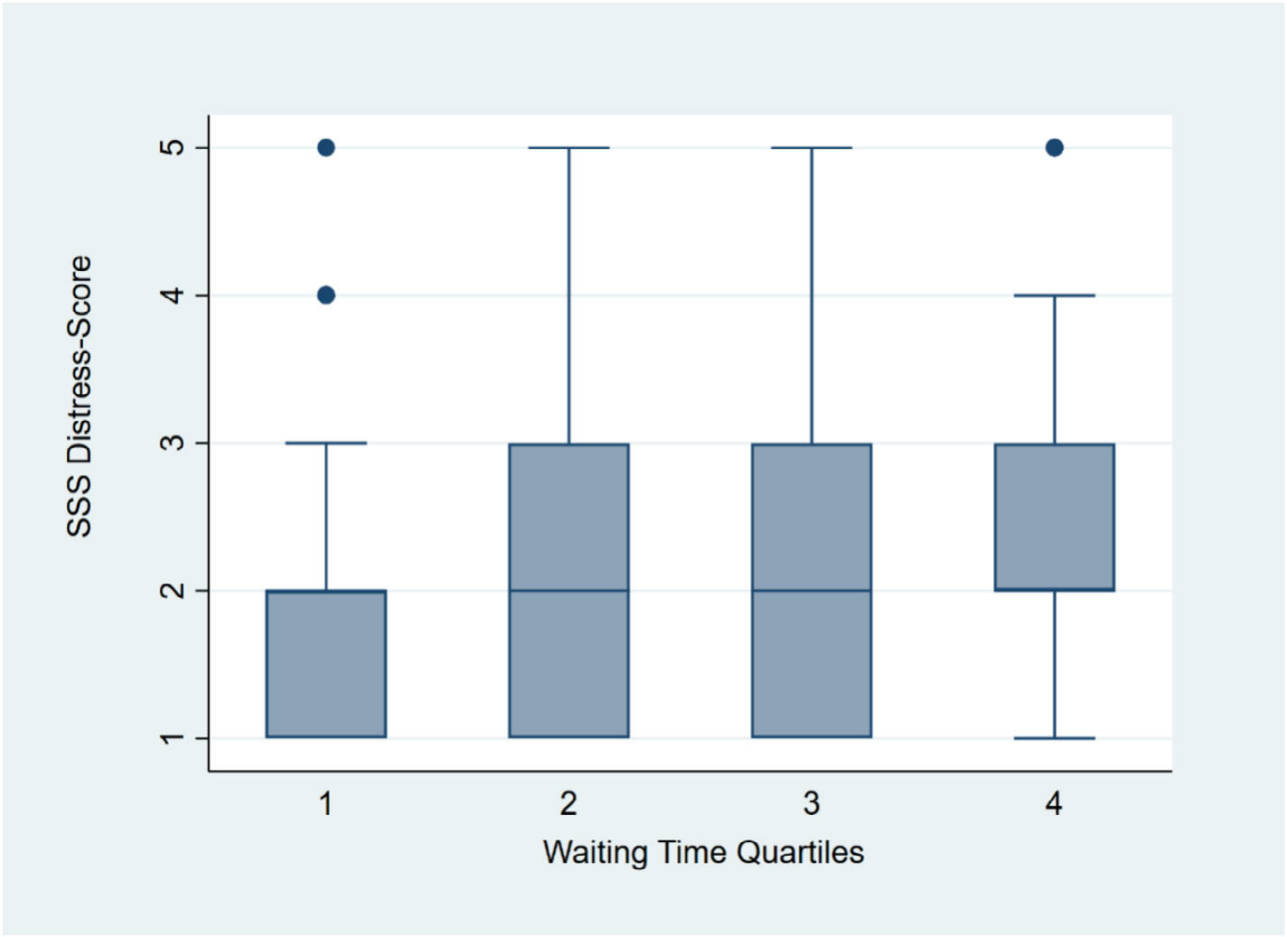
Distribution of severity symptom scale score across waiting‐time quartiles. Figure legend: boxplot of severity symptom scale (SSS) score across waiting‐time quartiles. Quartile 1 represents the shortest waiting times, and Quartile 4 the longest. Higher SSS scores indicate greater physical distress.

Longer waiting times were not significantly associated with the odds of experiencing moderate to very severe symptoms (SSS score ≥3) prior to surgery (OR 1.01, 95% CI: 1.00–1.02; *p* = 0.09). In linear regression using waiting‐time quartiles, patients in the third and fourth quartiles reported significantly higher SSS scores compared with those in the shortest waiting‐time quartile (*β* = 0.36, 95% CI 0.06–0.66, *p* = 0.02; *β* = 0.50, 95% CI 0.20–0.80, *p* = 0.001; Figure 1).

### 3.3 Subgroup analysis

In the subgroup of patients with postponed surgery, a higher proportion of patients undergoing non‐oncologic procedures reported psychological distress (NCCN ≥4: 57.8% vs. 41.7%, *p* = 0.05) and moderate to very severe symptoms (SSS ≥ 3: 48.2% vs. 10.9%, *p* < 0.001) compared with patients undergoing oncologic surgery, while no significant differences were observed for Global Mental or Physical Health T‐scores (Table 4). A comparative analysis of patient‐reported outcomes according to surgical postponement in oncological and non‐oncological surgery is shown in Table S1. In multivariable regression analyses adjusting for waiting time, patients with delayed oncologic surgery had significantly lower odds of moderate to very severe symptoms (SSS ≥ 3) compared with those undergoing delayed non‐oncologic surgery (OR 0.13, 95% CI 0.05–0.35, *p* < 0.001). In contrast, no significant difference was observed for psychological distress (NCCN ≥4) after adjustment (OR 0.59, 95% CI 0.31–1.14, *p* = 0.119; *data not shown*).

### 3.4 Postponement‐related complications

Among the 249 patients whose surgeries were postponed, 75 (30.1%) reported experiencing at least one complication during the waiting period. The most frequently reported complications were pain (12.9%), UTIs (11.7%), urinary retention (6.4%) and macroscopic haematuria (2.4%). Physician‐documented complications were recorded in 36 patients (14.5%), with UTI (10.9%) and urinary retention (2.0%) being most frequent (Table 2). Two patients experienced tumour progression, defined as histopathological upstaging following surgical delay: One patient with prostate cancer progressed from cT3 to pT4 and one patient with renal cell carcinoma from cT1a to pT3a disease.

**TABLE 2:** Patient‐reported reasons for surgery postponements and complications following postponement.

	Surgery postponed
Total	249 (100%)
Patient informed about reason for surgery postponement (*n*, %)	
*Yes*	140 (56.22)
*No*	67 (26.91)
*NA*	42 (16.87)
Patient‐reported reason for surgery postponement (*n*, %)	
*Institutional capacity constraints*	111 (44.58)
*Patient‐related factors*	8 (3.21)
*Health reasons*	21 (8.43)
*Surgery preparation incomplete*	12 (4.82)
Patient‐reported complication resulting from postponement (*n*, %)	
*Complication*	75 (30.12)
*No complication*	160 (64.26)
*NA*	14 (5.62)
Type of patient‐reported complication (*n*, %)	
*Pain*	32 (12.85)
*Urinary tract infection*	29 (11.65)
*Urinary retention*	16 (6.43)
*Macroscopic haematuria*	6 (2.41)
*Other*	19 (7.63)
Physician‐reported complication resulting from postponement (*n*, %)	
*Complication*	36 (14.46)
*No complication*	196 (78.71)
*NA*	17 (6.83)
Type of physician‐reported complication (*n*, %)	
*Urinary tract infection*	27 (10.84)
*Sepsis*	0
*Urinary retention*	5 (2.01)
*Macroscopic haematuria with necessary intervention*	0
*Tumour progression*	2 (0.80)

Abbreviations: NA, not available (missing data); SD, standard deviation.

**TABLE 3:** Patient‐reported psychological and physical burden according to surgical postponement.

	Overall	Surgery not postponed	Surgery postponed	*p*‐value
	*n* = 488	239 (48.98%)	249 (51.02%)	
NCCN distress thermometer* *NCCN < 4* *NCCN > = 4* *NA*	196 (40.16) 224 (45.90) 68 (13.93)	92 (48.17) 99 (51.83)	104 (45.41) 125 (54.59)	0.57
Symptom severity scale* *SSS < 3* *SSS > = 3* *NA*	266 (54.51) 147 (30.12) 75 (15.37)	137 (69.90) 59 (30.10)	129 (59.45) 88 (40.55)	**0.03**
Global mental health T‐score* (mean ± SD)	44.48 (5.45)	45.12 (5.29)	43.89 (5.55)	**0.03**
Global physical health T‐score* (mean ± SD)	42.97 (5.25)	43.03 (5.62)	42.91 (4.88)	0.45

*Note*: *Questionnaire data collection was conducted on the day of admission for surgery.

Abbreviations: NA, not available (missing data); SD, standard deviation.

**TABLE 4:** Subgroup analysis of patients with postponed surgery: comparison between delayed oncologic and delayed non‐oncologic surgery.

	Oncologic surgery postponed	NON‐oncologic surgery postponed	*p*‐value
	*N* = 52	*N* = 196	
NCCN distress thermometer* *NCCN < 4* *NCCN > = 4* *NA*	28 (58.33) 20 (41.67)	76 (42.22) 104 (57.78)	**0.05**
Symptom severity scale* *SSS < 3* *SSS > = 3* *NA*	41 (89.13) 5 (10.87)	88 (51.76) 82 (48.24)	**<0.001**
Global mental health T‐score* (mean ± SD)	43.60 (5.05)	43.99 (5.69)	0.46
Global physical health T‐score* (mean ± SD)	43.00 (4.72)	42.93 (4.91)	0.99

*Note*: *Questionnaire data collection was conducted on the day of admission for surgery.

Abbreviations: NA, not available; SD, standard deviation.

## 4 DISCUSSION

There is limited research on the psychological and physical impact of patients wait‐listed for elective urological surgery, particularly those with benign conditions. Our study aimed to address this gap by systematically evaluating symptom burden, mental and physical health status and delay‐related complications using validated patient‐reported outcome measures. This was particularly relevant in our region, where only two urology departments provide tertiary care for approximately 1.2 million inhabitants. Due to persistent staff shortages and reduced surgical capacity, patients often face substantial delays, with waiting times exceeding 30 weeks in postponed cases, underscoring the need to better understand the consequences of delayed care.

Several of our findings are noteworthy. First, shorter waiting times for surgeries for suspected or confirmed oncological diagnoses come at the expense of procedures for benign indications. Patients with a non‐oncological diagnosis were significantly more likely to experience postponements and waited on average 31 weeks for surgery. This finding supports the observed decline in elective urological procedures, mainly for benign conditions.^3^ While there is a substantial body of research on postponed therapy leading to tumour progression,^12^, ^13^ evidence on the effects of delayed treatment in benign urological conditions is limited.

Delayed surgical treatment of benign prostatic obstruction can lead to recurrent urinary retention, UTIs and a significant symptom burden, as demonstrated by the complications observed in the current investigation. Furthermore, urinary retention and delayed surgery after catheterization are associated with poorer long‐term postoperative outcomes.^14^ Long‐term catheterization leads to recurrent UTIs, discomfort and pain and has significant impacts on patients' social life and sexual health.^15^ Postponed HoLEP surgery has been associated with prolonged recovery from lower urinary tract symptoms and persisting complaints.^16^ For reconstructive surgeries as well, the risks of complications, such as severe recurrent UTIs or fistula formation, may increase with surgery delays.^17^ These observations show that even supposedly ‘benign’ conditions can result in a burden of symptoms that can significantly worsen with delayed treatment and place a considerable strain on patients. Together with the high rate of complications (30%) among patients whose surgery was postponed, these findings suggest the need to at least carefully monitor patients with prolonged waiting times.

In addition to physical consequences, prolonged waiting times may contribute to psychological distress, including anxiety, depression and other mental health challenges.^18^, ^19^ We demonstrated that patients with postponed surgery had significantly lower Global Mental Health T‐scores than those whose surgeries were not postponed. Pyrgidis et al. reported that postponement of elective sexual or reproductive health operations (e.g. external genitalia surgery in our cohort) impairs personal and sexual life.^20^ This is important, as untreated psychological distress can intensify physical symptoms and delay postoperative recovery.^21^ While extensive psychosocial distress was reported in patients with cancer facing surgery delays during the COVID‐19 pandemic,^19^ our findings show that patients with benign conditions were also psychologically impacted. The reason for this elevated psychological distress might be closely related to complications and persistent discomfort or pain due to patients' urological conditions as well as significantly longer waiting times.

Two patients in our cohort experienced tumour progression during the surgical waiting period. This underscores the potentially serious oncological consequences of surgical delays and highlights the need for timely intervention in high‐risk cases. Although this study did not systematically assess oncological outcomes, these cases illustrate a real risk that warrants further evaluation in dedicated, diagnosis‐specific cohorts.

We clarified that the most common reason for postponement was limited surgical capacity, primarily due to ongoing staff shortages. Most delays were not caused by poor preoperative planning or patient‐related issues, but rather by systemic constraints beyond the control of treating physicians. These insights have already contributed to improved capacity planning and patient triage. Multiple approaches have already been implemented to address limited operating capacities and the growing waiting lists and waiting times for elective surgery. We outsourced simple minimally invasive procedures (e.g., endourological procedures such as ureterorenoscopy and TURBT of small bladder tumours, surgery of the external genitals such as hydrocele resection and circumcision) to low‐volume hospitals. Similarly, in England, elective surgical hubs showed promising effects on elective activity in hospital trusts focusing on high‐volume, low‐complexity specialties.^22^ Triage systems to prioritize cases based on oncological risk and symptom burden have further been implemented at our department since 10/2024. Although prioritization does not necessarily reduce total waiting time for all patients, it may help ensure that those with the greatest clinical need are treated first. To bridge the waiting time, teleconsultations may also be offered, a concept that has already proven to provide interim relief in urological patients.^23^ However, transparent communication with healthcare teams about the situation might not be helpful for all patients. In our cohort, 56% patients were aware of the reason for postponement, most commonly a lack of operating room capacity.

Finally, systematic monitoring of waiting times, along with studies like this one, is essential to put pressure on policymakers and to promote sustainable strategies for improving the situation.

Several limitations of the present study should be noted. First, only a small proportion of patients admitted for elective surgery participated. While selection bias toward dissatisfied patients cannot be excluded, some may have declined due to frustration or disengagement, potentially skewing results in either direction. Second, most participants (70%) were retired, limiting conclusions about the economic impact of surgical delays in the working population. Third, only a small number of patients with urolithiasis were included due to outsourcing of these procedures. Fourth, this was a single‐centre study with a limited sample size. Fifth, although the NCCN Distress Thermometer is validated for patients with cancer or chronic illness, its use in benign, potentially curable conditions is less established. Finally, while two cases of tumour progression were observed, the study was not designed to assess oncological outcomes.

## 5 CONCLUSION

Patients with urologic conditions requiring surgical treatment in Styria are confronted with unacceptably long waiting times. Delayed elective urological surgeries are associated with increased symptom burden and psychological distress. These findings underscore the urgent need for health policy action to address staff shortages and to provide sufficient resources to ensure adequate surgical care.

## AUTHOR CONTRIBUTIONS

*Conception and design*: Iva Simunovic, Hanna Zurl, Marianne Leitsmann, SaschaAhyai and Almut Frank. *Acquisition of data*: Iva Simunovic, Julia Altziebler, Conrad Leitsmann, Lukas Scheipner, Johannes Mischinger, Klara Pohl, Anna Mangge and Gerhard Bachmaier. *Analysis and interpretation of data*: Iva Simunovic, Hanna Zurl, Marianne Leitsmann, Sascha Ahyai and Alexandra Weiss. *Drafting of the manuscript*: Iva Simunovic, Hanna Zurl, Marianne Leitsmann, Conrad Leitsmann and Klara Pohl. *Critical revision of the manuscript for important intellectual content*: Almut Frank, Gerhard Bachmair, Conrad Leitsmann, Sascha Ahyai, Johannes Mischinger and Lukas Scheipner. *Statistical analysis*: Hanna Zurl, Lukas Scheipner and Gerhard Bachmaier. *Administrative, technical, or material support*: Gerhard Bachmaier, Marianne Leitsmann, Hanna Zurl and Iva Simunovic. *Supervision*: Hanna Zurl and Marianne Leitsmann.

## DISCLOSURE

During the preparation of this work the authors used ChatGPT 4.o in order to language editing. After using this tool, the authors reviewed and edited the content as needed and take full responsibility for the content of the publication.

## CONFLICT OF INTEREST STATEMENT

The authors declare no conflicts of interest.

## Supporting information


**Table S1.** Comparative analysis of patient‐reported outcomes according to surgical postponement in oncological and non‐oncological surgery.

## Data Availability

All data are available from the corresponding author.
