# Supplementary material for: Impact of delayed elective urological surgery: A prospective observational study
Source: BJUI Compass. 2026 Apr 11;7(4):e70197. doi: 10.1002/bco2.70197 (PMC13069355; doi:10.1002/bco2.70197)
Supplement: Supplementary file 1 — Table S1. Comparative analysis of patient‐reported outcomes according to surgical postponement in oncological and non‐oncological surgery. [file BCO2-7-e70197-s001.docx]

**SUPPEMENTARY TABLE**

**Suppl. Table 1. Comparative analysis of patient-reported outcomes according to surgical postponement in oncological and non-oncological surgery**

| Suppl. table 1 | ONCOLOGIC SURGERY | SURGERY NOT POSTPONED | SURGERY POSTPONED | p-value |
| --- | --- | --- | --- | --- |
|  | **n=183** | **131 (71.58%)** | **52 (28.42%)** |  |
| NCCN Distress Thermometer*  *NCCN<4*  *NCCN>=4*  *NA* | 77 (42.08)  70 (38.25)  36 (19.67) | 49 (49.49)  50 (50.51) | 28 (58.33)  20 (41.67) | 0.31 |
| Symptom Severity Scale*  *SSS<3*  *SSS>=3*  *NA* | 130 (71.04)  23 (12.57)  30 (16.39) | 89 (83.18)  18 (16.82) | 41 (89.13)  5 (10.87) | 0.35 |
| Global Mental Health T-score*  (mean±SD) | 44.72 (5.58) | 45.17 (5.74) | 43.60 (5.05) | 0.10 |
| Global Physical Health T-score* (mean±SD) | 42.96 (5.26) | 42.94 (5.48) | 43.00 (4.72) | 0.88 |
|  | **NON-ONCOLOGIC SURGERY** | **SURGERY NOT POSTPONED** | **SURGERY POSTPONED** | **P-VALUE** |
|  | **n=304** | **108 (35.53%)** | **196 (64.47%)** |  |
| NCCN Distress Thermometer*  *NCCN<4*  *NCCN>=4*  *NA* | 119 (39.14)  153 (50.33)  32 (10.53) | 43 (46.74)  49 (53.26) | 76 (42.22)  104 (57.78) | 0.48 |
| Symptom Severity Scale*  *SSS<3*  *SSS>=3*  *NA* | 136 (44.74)  123 (40.46)  45 (14.80) | 48 (53.93)  41 (46.07) | 88 (51.76)  82 (48.24) | 0.74 |
| Global Mental Health T-score*  (mean±SD) | 44.36 (5.38) | 45.05 (4.71) | 43.99 (5.69) | 0.07 |
| Global Physical Health T-score* (mean±SD) | 43.00 (5.23) | 43.13 (5.81) | 42.93 (4.91) | 0.41 |
| **Questionnaire data collection was conducted on the day of admission for surgery. Abbreviations: SD= standard deviation, NA=not available (missing data)* | | | | |
